# A Cursory Glance at Tetanus

**Published:** 1891-07

**Authors:** G. Lenox Curtis


### A CURSORY GLANCE AT TETANUS.[1]

BY G. LENOX CURTIS, M.D., D.D.S.

My attention was accidentally directed to the subject chosen through having been asked for advice in a case of tetanus, after I had accepted your invitation to address you this evening.

A railroad employé had his hand crushed, and the case came under the care of the company surgeon. On Thursday morning last the patient was unable to open his mouth, and the surgeon, knowing that I had given the subject of tetanus some attention, asked for my opinion. After hearing his statement, I advised amputation of the arm above the line of demarcation, for the reason that, according to the description, the hand would have to go anyway, and that it would be well to attempt to save the patient's life by making sure of the entire elimination of the source of the tetanic trouble. I further suggested that if the surgeon wished to test the actual presence of tetanus, he could do so in a few hours by inoculating a mouse with matter from the crushed hand; that the poison would quickly kill it, and that then the exudate would show, under the microscope, the presence or absence of the tetanus bacillus.

This incident suggested the bringing of the subject before you. Not that tetanus is a disease which falls within the usual province of the dentist, but that it is sufficiently related to the territory over which his jurisdiction ordinarily extends to justify the bestowal of some attention upon it; particularly as dental operations, carelessly performed, may readily induce the dread symptoms of lockjaw. Cases have been recorded, for instance, of tetanus directly traceable to the irritation set up by replanted teeth; and although, so far as I am aware, there are no reported instances of its occurrence following the more modern implantation procedure, its appearance among the train of events consequent upon that operation would have been no surprise to many conservative practitioners.

It is well to get outside occasionally of the beaten track of the ordinary round of duties. Constant concentration of the faculties upon any given pursuit will, it is true, give one a mastery not otherwise attainable, but it may be questioned whether pre-emi-

---

[1] Read before the New York Odontological Society, Tuesday evening, April 21, 1891.

nence so gained is worth the cost. Just in proportion as one devotes himself to a single end will he lose the capacity for interest in and enjoyment of those things which do not assist in its accomplishment. Narrowing the field of knowledge and experience begets bigotry, the natural and logical child of narrow-mindedness, a condition not readily reconcilable with any legitimate claim for professional attainments. It is not only proper, but absolutely essential for one who hopes to achieve the highest honors of his profession to keep in touch not merely with related sciences, but also with the general progress of the age. To bring the application home to our own profession, it is undeniably true that, other things being equal, the broader the culture the better the dentist.

In presenting the subject of tetanus this evening there will be no attempt at orderly arrangement or exhaustive treatment. Time only permits the jotting down of thoughts as they occur. Sufficient will have been accomplished if your interest is awakened in even a slight degree.

The derivation of the word tetanus is Greek, and its literal meaning is "stretched," by which is accurately described the condition of the muscles affected. Notwithstanding that the disease has been long recognized and much written upon, there is even yet considerable doubt as to its pathology. Two forms are recognized, traumatic and idiopathic, the former arising from a wound upon any part of the body, though more commonly following injuries to the extremities, and the latter where there is no apparent lesion of the skin. It is perhaps an open question whether the latter, although exhibiting the same or nearly identical symptoms, is a true tetanus.

*Trismus nascentium* was formerly generally classed with tetanus, and even to-day there are many who recognize no essential difference between it and true traumatic tetanus. A very exhaustive paper on trismus was printed in the *American Journal of the Medical Sciences*, in 1884, from the pen of Dr. J. F. Hartigan, of Washington, D. C. In this paper, after quoting the opinions of most of the prominent writers on the subject for fifty years or more, he gave his own experiences, covering two hundred and twenty-nine cases, in many of which he had performed autopsies. His conclusions were as follows:

"Now, with regard to the identity claimed between this disease and tetanus in the adult, it will be apparent that the statistics presented do not confirm this view. It is also disproved by the comparisons of Dr. Sutton and others previously given. Holmes mentions 277 cases of traumatic tetanus, of which only 130, or forty-seven per cent., were seized within ten days after the injury. I was enabled to learn the time of seizure from birth in 209 of my cases of trismus, of which number 189, or ninety per cent., were under ten days; while of the 88 reported by Stadtfeldt only one survived the tenth day. Matuszynski's 25 were all seen before the ninth day, and 107 of the 114 of Clarke's died before the ninth day.

"In a table of 327 fatal cases of tetanus in the adult, also reported by Holmes, 79, or twenty-four per cent., succumbed within two days. I ascertained the average duration of sickness in 207 of my cases to be fifty hours, of which number, 165, or about eighty per cent., lived two days and under, and 86, or forty-one and six-tenths per cent., lived one day and under.

"Another point in refutation of this claim of identity is the fact that in the tetanus of the adult febrile excitement is not essential, and if present is only secondary. Indeed, O'Beirne states that of 200 cases observed by him, not one was accompanied by fever. In every instance of trismus nascentium, where I took the temperature when the disease was at its height, I found an elevation, one case that I saw with Dr. Shadd, of the Freedman's Hospital, reaching 105.4°.

"Neither are the post-mortem appearances alike. The brain, medulla, and cord have been found in various conditions in the adult, but very rarely if ever have coagula been seen, although universally present in the infant. The similarity of these diseases in other respects I admit, but their etiological relations I deny. The principal basis of identity claimed is *that* trauma necessarily inflicted in the cutting of the cord at birth, irritating dressings, etc., to navel, which is supposed to correspond with the wound in the adult."

It should perhaps be added that Dr. Hartigan's experience and observation confirmed the opinion of Sims, that *trismus nascentium* was not at all related to the cutting of the cord, but was caused by dislocation of the bones of the head, occurring sometimes at birth, but more commonly through unskilful or careless handling afterwards, and producing brain-pressure by the displacement. Where the trouble was recognized in time, and the replacing of the bones was possible, a cure resulted. Certain it is that in the infant under five months all the symptoms of trismus can be produced in a few minutes by dislocating these bones at the occipito-parietal suture.

Traumatic tetanus has its origin in a wound of the surface of the body. As to the manner in which the characteristic symptoms are set up there is no positive knowlédge. By some it is regarded as a reflex action, and undoubtedly it often bears this appearance. Others claim that it is caused by direct impingement upon the nerves leading to the wounded part. The latest theory upon the subject is that it is caused by a bacillus, and a so-called tetanus bacillus has been isolated. Since this theory was promulgated, the bacillus described has been seen by many other observers. Indeed, it is found as a constant accompaniment when searched for. In 1888, M. Bossano showed that the disease was bacterial in its origin, but that the transmission of the virus by inoculation through several animals brought about its attenuation and ultimately rendered it inert as regards the production of the disease. Personally I believe the bacillus theory is the true explanation; that tetanus comes from inoculation, and that otherwise the disease is not tetanus.

As to the source of the inoculation, it has been shown that most garden dirt—land that has been worked—is infected with this particular bacillus. Soil from different parts of the world was used in experiments on mice and guinea-pigs, and in nearly every instance it was found to be sufficiently charged with tetanus bacilli to produce death. It was also found that the more organic matter the specimen of soil contained, the greater the number of bacilli. We have here perhaps a clue to the universal locality of tetanus. Rusty nails, to which are commonly and correctly ascribed superior powers in the causation of lock-jaw, are also likely to be infected from the same wide-spread source. What wonder, then, that wounds exposed to such common opportunities of infection should be inoculated with the fatal poison?

For a statement of the symptoms of tetanus I can perhaps do no better than to quote Macnamara:

"Tetanus almost invariably commences in rigidity of the muscles of expression. In the course of a few hours the muscles of mastication and of the head, neck, and back become involved so that the patient experiences difficulty in opening his mouth, or in moving his head from side to side; and deglutition is impeded by spasmodic contraction of the pharynx. The rigidity of one or more of the groups of muscles above referred to is constant throughout the whole course of the disease; but in addition to this, from time to time these muscles are thrown into the most frightful spasms; in this way the patient's body is sometimes bent like a bow, the whole weight of the trunk being supported on the back of his head and heels. The abdominal and thoracic muscles are also implicated, and hence the patient's belly is tense and hard, and the walls of his chest expand imperfectly in the effort of breathing. The muscles of the arms and legs are often extremely rigid and convulsed in a most violent manner; they are the seat of terrible pain. The interval between the paroxysms of spasm of the affected muscles is very uncertain; sometimes the cramps last only for a few seconds, at other times for five and even ten minutes. The most dangerous cases of tetanus are evidently those in which the muscles of respiration are principally involved, for death is generally caused in this disease by the interference with the respiratory process, the chest being, as it were, compressed in a vice. In consequence of the condition of the muscles of the neck and thorax the sick person is unable to speak, but his intellect generally remains clear up to the last, nor are the other functions of his body materially deranged. The patient suffers much from hunger and thirst, which he is unable to alleviate; and, above all, he longs for sleep, which is frequently denied him in consequence of the recurring spasms. The surface of the skin is bedewed with perspiration and the pulse rises and falls with the intensity of the spasms and the duration of the disease."

Tetanus runs a definite course. Sometimes it kills in a few hours, but the greater number of victims die from the seventh to the eleventh day after the commencement of the disease. If they survive the twelfth day, a cure usually occurs.

As to the treatment, thorough understanding of that is perhaps not so important to us as dentists as is a full and accurate knowledge of the diagnostic signs of its presence, more especially as its first expression is found in the face, and one of its most serious phases in the locking of the jaws. In general, it may be said that there is really but little to be done in the way of internal medication. Most of the drugs which have been prescribed to arrest the progress of the disease or allay the violence of the spasms fail to accomlish recognizable results, unless pushed to an extreme which threatens grave and scarcely justifiable complications. The first indication should be the thorough cleansing of the wound, which should be placed at once in an aseptic condition and kept so. If such a procedure were promptly instituted and skilfully carried out in all cases of wounds, I think it extremely probable that traumatic tetanus would cease to find victims. Professor Garretson regards phenol sodique as a sheet-anchor for dressings as a prophylatic against tetanus.

In any event, when tetanus is developed, it is a safe plan to bend every energy towards keeping the patient alive until the malady has spent its force; more especially because of the well-ascertained definiteness of the course of the disease, although its duration is uncertain, so that, if vitality can be preserved through the terrible agony that must be endured under the double deprivation of natural sleep and normal nourishment, there need be but little apprehension of fatal consequences. Under the conditions presented this is no easy task. The administration of food and the procuring of sleep are alike difficult and uncertain. As food, milk is, of course the staple, and as a sedative hydrate of chloral will perhaps produce as good results as any.

If the germ theory be finally accepted, surgical interference, where practicable, will be indicated, and fortunately the great majority of wounds which eventuate in tetanus are so situated that amputation of the affected part is entirely feasible. Cases are on record, also, where division of the principal nerve, or in other cases stretching of the nerve leading from the wound, has completely stopped the progress of tetanus.

Perhaps enough has been said to attract your attention to this formidable disease, which has been the object of the preparation of this brief paper. Much could have been said had the purpose been a didactic exposition of the subject; but to suggest a thought upon what may readily occur in the practice of any of us was all that was sought. Fortunately for our peace of mind, although the records show a few cases, tetanus as a sequel of dental operations is exceedingly rare. Professor Garretson has never met with a case; nor have several other gentlemen to whom I have spoken. Let us hope that our experience in the future may be equally fortunate. If what has been said here has the effect to make even one practitioner more careful in the performance of such operations as have been proved to occasionally produce tetanus, I shall be most glad of having been the instrument of so much good.

